# Current advances and future perspectives on the functional roles and clinical implications of circular RNAs in esophageal squamous cell carcinoma: more influential than expected

**DOI:** 10.1186/s40364-022-00388-y

**Published:** 2022-06-07

**Authors:** Chenxi Ju, Jing He, Chang Wang, Jinxiu Sheng, Jinlin Jia, Dan Du, Hongle Li, Mingxia Zhou, Fucheng He

**Affiliations:** 1 Department of Medical Laboratory, The First Affiliated Hospital of Zhengzhou University, Zhengzhou, 450052 China; 2 Department of Breast Surgery, The First Affiliated Hospital of Zhengzhou University, Zhengzhou, 450052 China; 3 Department of Molecular Pathology, The Affiliated Cancer Hospital of Zhengzhou University, Zhengzhou, 450008 China; 4 Department of Gastroenterology, The First Affiliated Hospital of Zhengzhou University, Zhengzhou, 450052 China

**Keywords:** circRNA, Diagnosis, Treatment, Esophageal squamous carcinoma (ESCC)

## Abstract

Esophageal squamous cell carcinoma (ESCC) is one of the most aggressive gastrointestinal cancers with high incidence and mortality. Therefore, it is necessary to identify novel sensitive and specific biomarkers for ESCC detection and treatment. Circular RNAs (circRNAs) are a type of noncoding RNAs featured by their covalently closed circular structure. This special structure makes circRNAs more stable in mammalian cells, coupled with their great abundance and tissue specificity, suggesting circRNAs may present enormous potential to be explored as valuable prognostic and diagnostic biomarkers for tumor. Mounting studies verified the critical roles of circRNAs in regulating ESCC cells malignant behaviors. Here, we summarized the current progresses in a handful of aberrantly expressed circRNAs, and elucidated their biological function and clinical significance in ESCC, and introduced a series of databases for circRNA research. With the improved advancement in high-throughput sequencing and bioinformatics technique, new frontiers of circRNAs will pave the path for the development of precision treatment in ESCC.

## Introduction

Esophageal carcinoma is an intractable problem worldwide for human health because of its high morbidity and poor prognosis. According to the epidemiological data in 2020, there will be about 604,000 new cases of esophageal carcinoma and 544,000 deaths globally, ranking seventh in the incidence rate and sixth in mortality of cancer overall [1]. Esophageal carcinoma is classified into esophageal squamous cell carcinoma (ESCC) and esophageal adenocarcinoma (EAC) based on the histological types. The two subtypes exhibit not only different etiologies and pathogenesis but also noticeable geographic differences. Most cases of EAC occur in developed countries, while ESCC incidence accounted for more than 90% in Asia and sub-Saharan Africa [1]. It has been revealed that ESCC was associated with environmental factors including smoking, alcoholism and genetic mutation [2]. Owing to lacking early specific symptoms, an enormous number of ESCC patients were diagnosed with the advanced stage or distant metastasis, leading to a dismal prognosis. Although the application of new tumor markers and advanced treatment strategies such as targeted therapy and immunotherapy, the 5-year survival rate of ESCC is still poor. Even after surgical resection, patients often have local recurrence or distant metastasis. Therefore, it is urgent to figure out the molecular mechanism of occurrence and metastasis of ESCC and ascertain more specific and compelling effective biomarkers for screening, diagnosis, and prognostic monitoring.

Recently, circular RNAs (circRNAs) in the field of the tumorigenesis and development of tumors have attracted much attention from researchers. As a novel type of non-coding RNAs (ncRNAs), circRNAs are covalently closed loop structures without 5’ caps and 3’ tails, which makes them more stable and RNA exonuclease resistance. However, when circRNAs were first discovered in the virus in 1976 [3], only a few of circRNAs were detected, and they were considered as a byproduct of erroneous splicing events without biological function [4]. Until the widespread use of high-throughput RNA sequencing (RNA-seq) and bioinformatics algorithms, significant advancement has been made in the research of circRNAs. Recent studies have confirmed that circRNAs have an essential role in regulating gene expression at the level of transcription and post-transcription, modifying cell proliferation, differentiation and apoptosis, and mediating cell immunoreaction [5] as well tumorigenesis, development, drug resistance, metastasis in multiply cancers, including ESCC. Herein, we summarize the biogenesis, biological function and molecular mechanisms of circRNAs in ESCC tumorigenesis and further predict its possible implications in ESCC diagnosis and treatment.

### Biogenesis of circRNAs

**Fig. 1:**
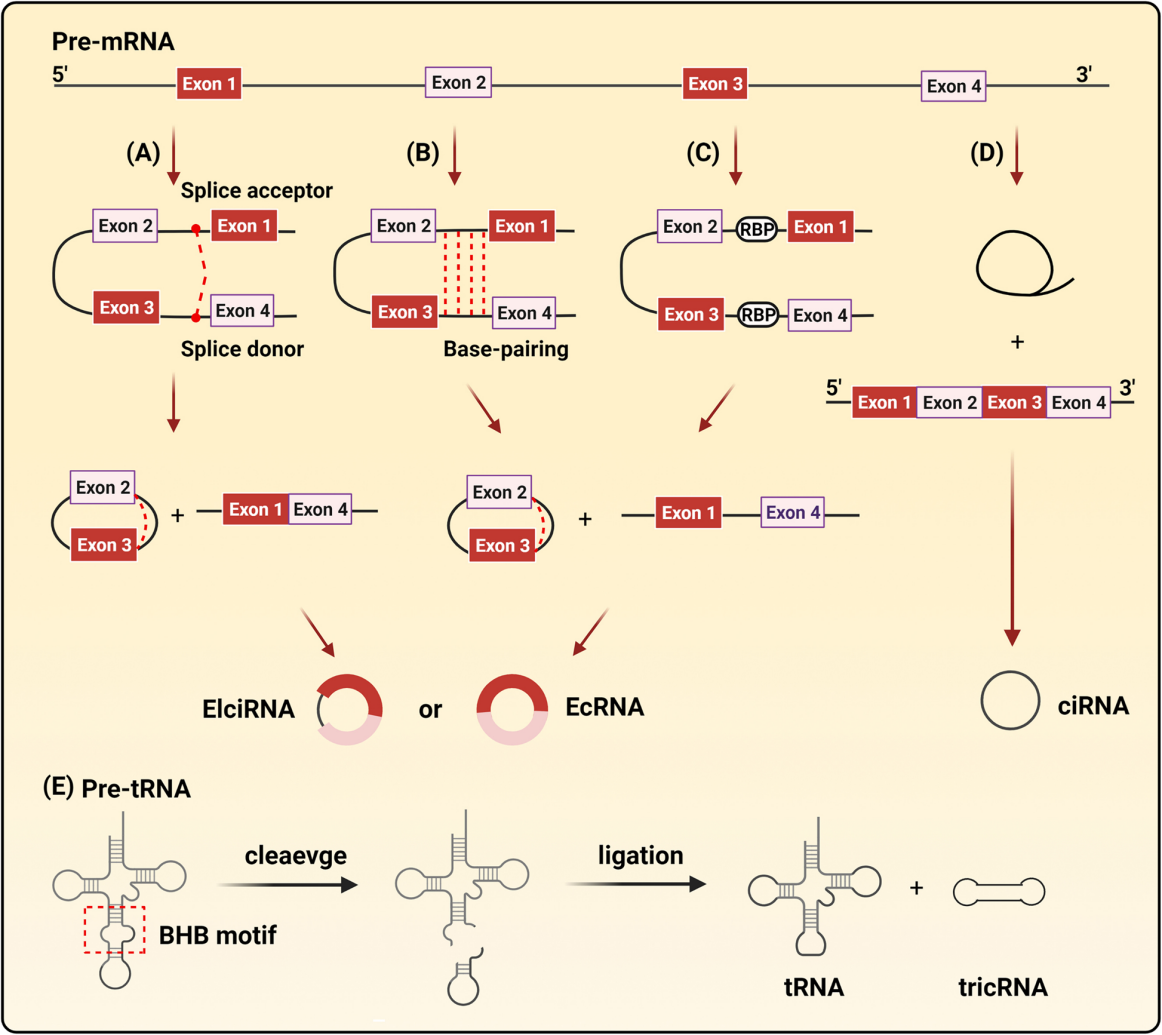
Biogenesis of circRNAs. **A** lariat-driven circularization: the folding of pre-mRNA caused it to form lariat structure which are generated from the link of 5’ splice acceptor and 3’ splice donor. Furthermore, the lariat structure conduct internal splicing of introns to form EIciRNA or EcRNA; **B** intron-pairing-driven circularization: pre-mRNA contained abundant complementary sequence in introns flanking the exons can connect to form EIciRNA or EcRNA by base pairing; **C** RBPs driven circularization: intronic motifs flanking exons have RBPs binding sites which can interact with RBPs and induce circularization, in this process, EIciRNA or EcRNA can be produced; **D** intron cyclization: pre-mRNA conduct internal splicing to remove introns and generate mature mRNA. Some spliced introns can circularization to form ciRNA; **E** the formation of tricRNAs: induced by tRNA splicing endonuclease (TSEN) which can recognize bulge-helix-bulge (BHB) motif, pre-tRNA can undergo internal splicing to generate tricRNA and tRNA

Most circRNAs are synthesized from precursor mRNAs (pre-mRNAs) through back-splicing and catalyzing by RNA polymerase II. It has been verified that back-splicing are competed with canonical pre-mRNA splicing [6], suggesting that circRNAs can exert biological effects through mediating pre-mRNA transcription. Compared to linear RNAs, circRNAs cyclization requires the link of 5’ and 3’ end, which is the leading trait of circRNAs. Most present research of circRNAs are based on this trait. According to the components, circRNAs are classified into three groups: exonic circRNAs (EcRNAs), exon–intron circRNAs (EIciRNAs) and circular intronic RNAs (ciRNAs). Several hypothetical models are proposed to depict the generation of circRNAs: 1) Lariat-driven circularization: Alternative splicing and the ligating of 3’ splice acceptor site and 5’ splice donor site form a lariat whose restricted structure facilitates circularization [7]. 2) Intron-pairing-driven circularization: Circularization relies on the complementary base pairing of different flanking introns, including ALU repeats [7]. 3) RNA binding proteins (RBPs) driven circularization: RBPs bind with the RBPs binding sites on the flanking sequence, and these two RBPs can promote circularization by bringing the splice site close [8]. 4) CiRNAs, synthesized from introns, are newly discovered in human cells. Circularization of ciRNAs requires a consensus motif containing a 7-nt GU-rich element near the 5’ splice site and an 11-nt C-rich element close to the branchpoint site to escape debranching [9]. Additionally, a few of circRNAs are generated from the splicing of precursor tRNAs (pre-tRNAs). This processing of such tRNA intronic circular RNAs (tricRNAs) requires tRNA splicing endonuclease (TSEN) complex to recognize bulge-helix-bulge (BHB) motif, and the introns that have been clipped are cyclized (Fig. 1) [10].

CircRNAs biosynthesis was reported to be associated with a variety of factors. The biogenesis of circRNAs is affected by the transcription rate of circRNA-producing genes. Cells with a high transcription rate normally own a higher expression level of circRNAs in neurons than those with slow transcription elongation speed [11]. RNA with different pairs of inverted complementary regions contains competitive base-pairing. RNA pairing across flanking introns facilitates circRNAs formation, while RNA pairing within individual intron restrains circRNAs formation [12]. Some proteins perform key function in the biogenesis of circRNAs. For instance, muscleblind (MBL) can enhance the production of circMbl by interacting with its binding sites in the intronic sequences flanking of pre-mRNA [6]. NF90/NF110 can target and stabilize transient dsRNAs formed by base-pairing of flanking introns and facilitate back-splicing [13]. Quaking (QKI) protein was identified to regulate circRNAs biogenesis in epithelial-to-mesenchymal transition (EMT) by targeting binding sites flanking circRNA-forming exons in linear RNA [8].

### Functions of circRNAs

**Fig. 2:**
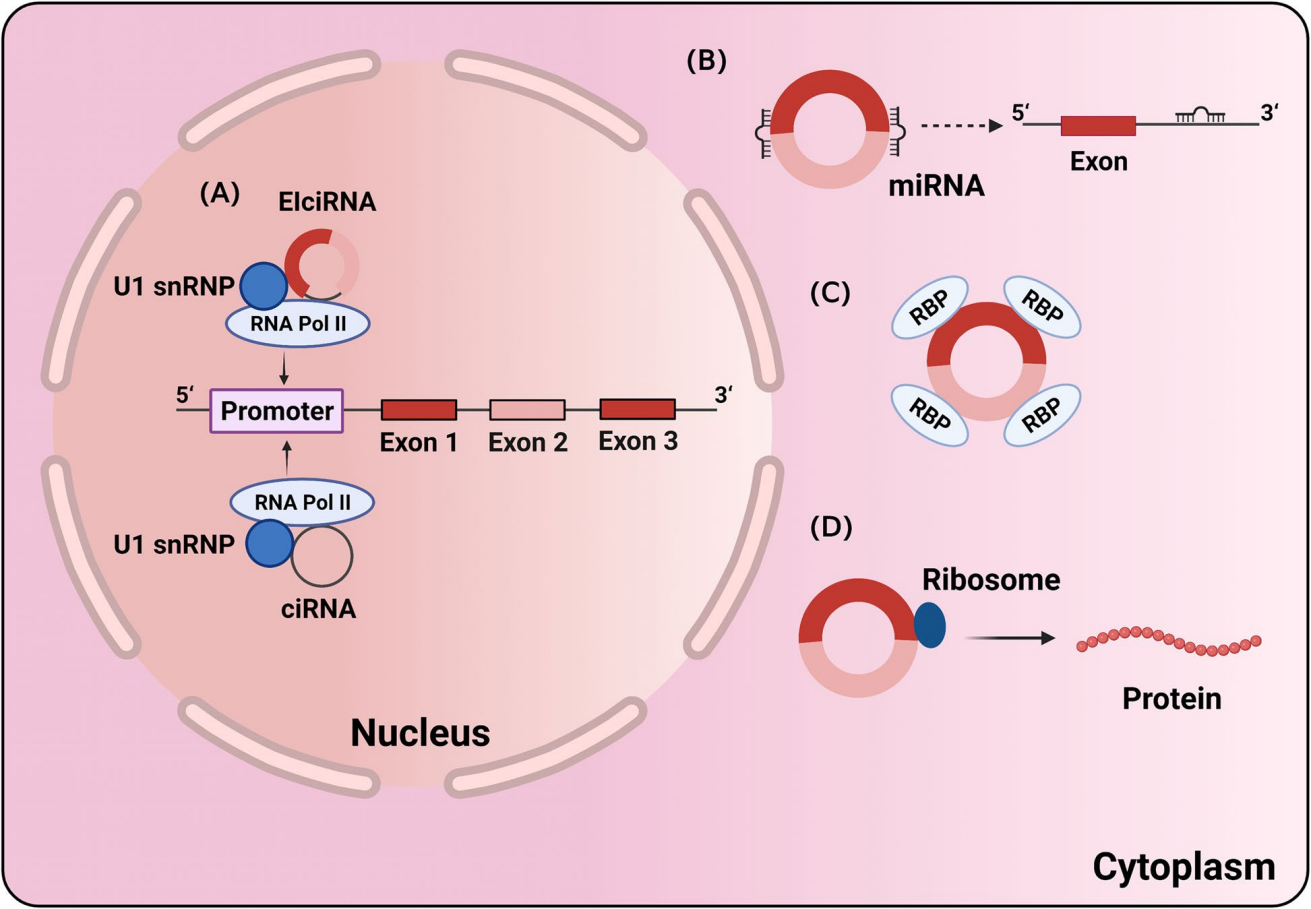
Function of circRNAs. **A** EIciRNA and EcRNA can interact with U1 snRNP to regulate host gene transcription in nucleus; In cytoplasm, (**B**) circRNAs act as miRNA sponge to reduce the function of miRNA; **C** circRNAs can bind and sequester proteins; **D** circRNAs can be translated into polypeptide

Through different mechanisms, circRNAs are involved in multiply process in various diseases. There are at least four functions of circRNAs which have been effectively proved by a number of studies (Fig. 2).

### Serving as miRNA sponge

MicroRNAs (miRNAs) could directly bind to miRNA response elements (MREs) of target mRNAs by base-pairing manner, inducing mRNA degradation and translation inhibition. Salmena et al. proposed competitive endogenous RNA (ceRNA) hypothesis that miRNAs could interact with all types of RNA contained with MREs, thus resulting in a competitive combination between miRNAs and other types of RNA and creating a ceRNA interaction network [14]. CircRNAs also hold numerous miRNAs binding sites, therefore, they can sponge miRNAs to relieve the suppression of miRNA underlying its target genes, serving as miRNAs sponges. For example, Thomas et al. found a circRNA highly expressed in human and mouse brain, named as ciRS-7, harboring 73 conserved binding sites for miR-7. Besides, they also proved another circRNA generated from the sex-determining region Y (Sry), which contains 16 binding sites for miR-138 [15]. This research is the first to show the potential of circRNAs to be miRNA sponge. After that, a series of researches have demonstrated the universality of this function. “miRNA sponge” has become the classical model of circRNAs function. However, it has long been controversial. A recent study found the high expression of ciRS-7 existed in colon cancer stromal cells rather than cancer cells, while miR-7 was only expressed in cancer cells. Therefore, the correlation between ciRS-7 and miR-7 target gene cannot be explained by ceRNA function [16]. More compelling evidence is needed to prove the exact mechanism of this action.

### Regulation of gene transcription

Although most circRNAs located in the cellular cytoplasm and predominantly function as miRNA sponges, EIciRNAs and ciRNAs expressed in the nucleus contain few miRNAs binding sites and have been identified to engage in the process of parental genes regulation. For instance, circEIF3J and circPAIP2 can enhance the transcription of their parental genes in cis through forming EIciRNA-U1 snRNP complexes and further interacting with Pol II transcription complex at the promoters of parental genes [17]. Similarly, Zhang et al. uncovered that ci-ankrd52 and ci-SIRT7 are able to gather in the transcriptional sites to modulate elongation Pol II complex, thus acting as positive cis-regulators of theirs host gene transcription[9]. Overall, these intron circRNAs can act as transcriptional regulators to positively regulate the transcription of their parental genes via modulating RNA pol II activity.

### Translation into proteins

Although circRNAs were initially classified as non-coding RNAs, most circRNAs are composed of exons and harbor binding sites of ribosomes, indicating they own the potential to be translated into proteins. Even lacking 5’cap and associated cap-binding protein factors, circRNAs could recruit ribosomal 40S subunits to initiate the translation through a special sequence in 5’ non-coding region which was termed as internal ribosome entry site (IRES) [18]. Such IRES element was firstly discovered in poliovirus [19] before in eukaryotic mRNAs [20, 21]. Chen et al. proved synthetic circRNAs also recruit 40S ribosomal subunit to encode peptides after inserting IRES element in the upstream of the initiator AUG codon [22]. Also, there is accumulating evidence that circRNAs include extensive N6-methyladenosine (m6A) modification [23], which can be an IRES to drive translation initiation in a cap-independent way. This process requires the participation of m6A reader YTHDF3 and translation initiation factors eIF4G2 and eIF3A [24]. Circ-ZNF609 was revealed to have the potential to encode a protein. Its portion of UTR in upstream of promoter serve as IRES recruiting ribosomes after a splicing event [25]. Besides, circPPP1R12A can be translated into a functional protein circPPP1R12A-73aa, activating the Hippo-YAP signaling pathway to accelerate colon cancer progression [26]. Collectively, the coding potential of circRNAs has been identified in various cancers, such as glioblastoma, breast cancer, bladder cancer [27]. Detailed mechanism still warrants further study.

### CircRNAs bind and sequester proteins

Several pieces of evidence have shown that circRNAs bind specific proteins to arrest their functions. In some cases, circRNAs can act as “protein sponge” to regulate gene expression at both transcriptional and translational level. For example, circACTN4 binds to far upstream element binding protein 1 (FUBP1) and thus preventing FBP interacting repressor (FIR) from interacting with FUBP1 to lower MYC transcription, resulting in tumorigenesis and metastasis of breast cancer [28]. Likewise, circPABPN1 can integrate with HuR and restrain the function of HuR. HuR serves as a regulator in gene expression and can equally mediate the translation of PABPN1 mRNA [29]. In addition, some circRNAs can combine with proteins to block their transmembrane process. A good example is that circ-Foxo3 binds to the cell cycle proteins CDK2 and cyclin-dependent kinase inhibitor 1 (CDKN1A, also known as p21^WAF1/CIP1^) to form a ternary complex, thus extinguishing the effect of CDK2 and blocking cell cycle progression [30]. Similarly, Du et al. verified the mechanism of circLPAR3 in inhibiting cellular senescence, which was enabling by interacting with anti-senescence proteins ID1 and E2F1, anti-stress proteins FAK and HIF1α to lead to their retain in the cytoplasm and arrest their functions of anti-senescence [31].

### Public database for circRNAs research

Recently, the research on circRNAs becomes an emerging hot spot, surging online databases have been developed to meet the increasing requirements of information on circRNAs. Here, we reviewed the circRNAs online database and their usage to provide suggestions for future research (Table 1). CircBase provides comprehensive circRNAs information, including genomic position, length, the circular junction site, and so forth [32]. Circ2Traits [33] and circRNA disease [34] store a large number of disease-associated circRNAs and their annotation. The circRNA-miRNA-mRNA interaction network could be predicted in CircInteractome [35] and CircNet [36]. Besides, CircInteractome [35] can be used for divergent primers and siRNA design as well as RBP prediction. The information about the transcriptional regulation of circRNAs can be obtained from TRCirc [37]. ExoRBase [38] is a database of exosome-derived ncRNAs in human blood. Circbank [39] proposed a standardized nomenclature for circRNAs facilitated circRNAs information acquisition. Overall, these databases provide applicable bioinformatics tools for circRNAs research in the tissue development and human diseases.

**Table 1:** Databases for circRNAs research

Database	Function	Website	Refs
CircBase	CircRNAs annotation	www.circbase.org	[32]
Circ2Traits	A database stores potential disease association of circRNAs and miRNA-circRNA-mRNA-lncRNA interaction network	gyanxet-beta.com/circdb	[33]
circRNA disease	Provides information of circRNAs associated with disease	cgga.org.cn:9091/circRNADisease	[34]
CircInteractome	Predicts binding sites of RBPs and miRNAs on circRNAs and primer design, siRNA design	circinteractome.nia.nih.gov	[35]
CircNet	CircRNA identification and predicts circRNA-miRNA-mRNA interaction	circnet.mbc.nctu.edu.tw	[36]
starBase v2.0	Predicts other ncRNAs (e.g., lncRNAs, circRNAs) from miRNA-mediated regulatory networks	starbase.sysu.edu.cn	[40]
CSCD	Identifies cancer-specific circRNAs and predicts cellular localization, miRNA-circRNA interaction, RBP and ORF of circRNAs	gb.whu.edu.cn/CSCD	[41]
circRNADb	CircRNAs annotation predicts protein-coding potential and corresponding protein features	reprod.njmu.edu.cn/circrnadb	[42]
TRCirc	Obtains transcriptional regulation information about circRNAs	www.licpathway.net/TRCirc	[37]
Lnc2Cancer 3.0	Cancer-related lncRNAs and circRNAs annotation	www.bio-bigdata.net/lnc2cancer	[43]
CIRCpedia v2	Annotation and expression analysis of circRNAs	www.picb.ac.cn/rnomics/circpedia	[44]
deepBase v3.0	CircRNAs annotation includes expression, prognosis and functional predictions	rna.sysu.edu.cn/deepbase3/index.html	[45]
Circbank	Explored a standard nomenclature, predicts miRNAs binding, circRNAs conservation and protein-coding potential	www.circbank.cn	[39]
exoRBase	Exosome-derived ncRNAs annotation	www.exoRBase.org	[38]
ExoceRNA atlas	A database contains tumor-related circRNAs in blood exosomes and corresponding mRNA-miRNA-circRNA interaction network	www.exocerna-atlas.com/exoceRNA	[46]

### CircRNAs in ESCC

**Fig. 3:**
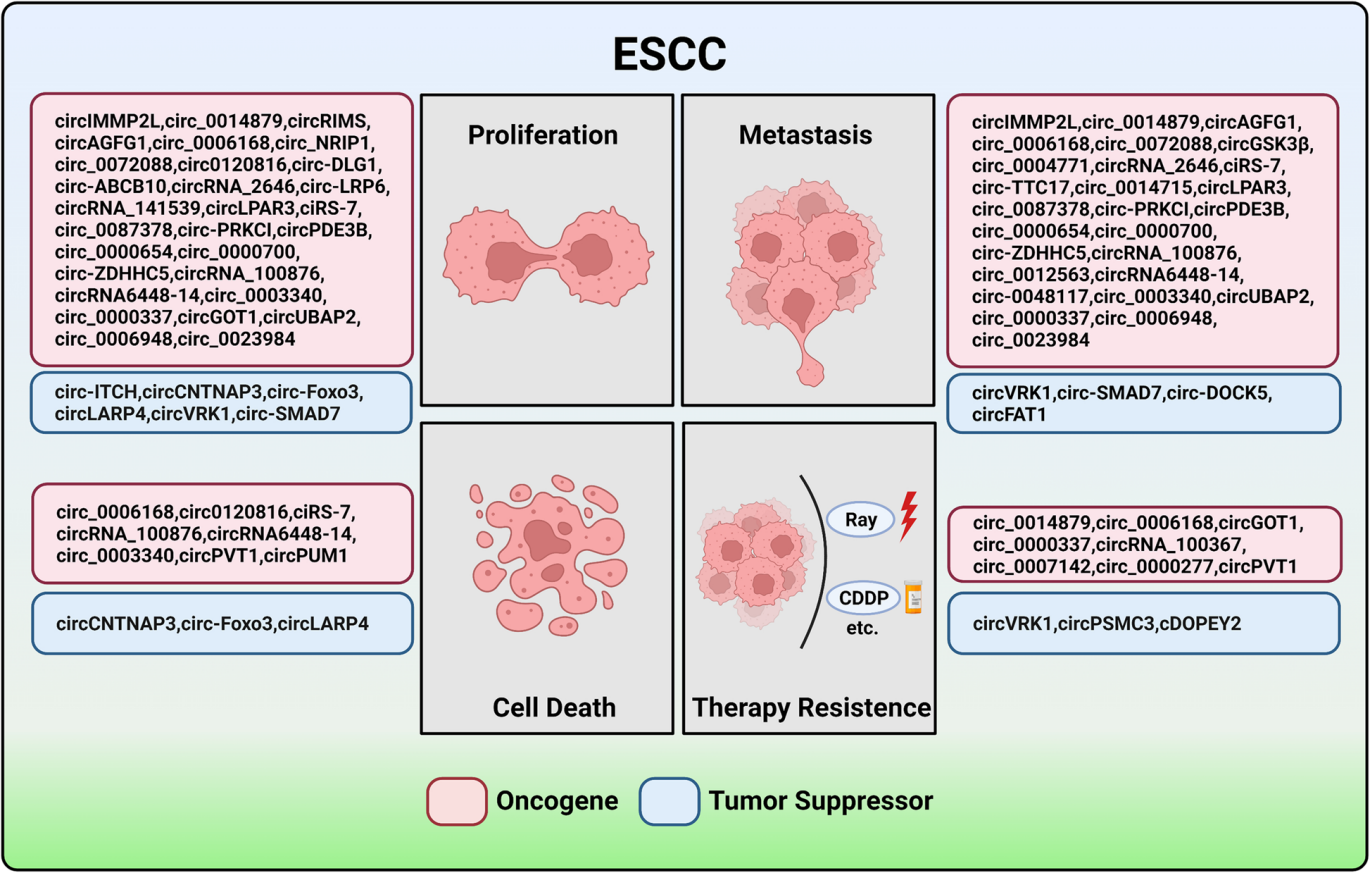
Summary of the function of circRNAs in ESCC. CircRNAs can participate in the origin and development of ESCC, including cell proliferation, cell death, migration and therapy resistance

There are multiply aberrant circRNAs in the oncogenesis and progression of ESCC, a significant percentage of which have been demonstrated to involve in various malignant biological phenotypes, such as cell proliferation, death, metastasis, drug resistance (Fig. 3). Below we summarize the most recent studies on aberrantly expressed circRNAs in ESCC, as well as their function, related mechanism underlying ESCC progression, and potential roles in tumor diagnosis and treatment (Table 2).

### Dysregulated expression of circRNA in ESCC

Emerging studies have reported the dysregulated expression of circRNAs between ESCC tissues and para-carcinoma tissues, suggesting circRNAs were the initiators in ESCC. This was largely accomplished by the development of high-throughput screening technology. CircRNA microarray analysis and RNA-seq are primarily used in circRNAs high-throughput screening. Unlike linear RNAs, the structural uniqueness and low content of circRNAs put forward new challenges. Circular junction sequence analysis is widely used in circRNAs identification. CircRNA microarray analysis just uses the principle of the specific integration of circRNA junction probe and circRNA junction sites to accurately detect the expression of circRNAs in tissue or plasma samples. However, circular junction reads obtained by RNA-seq are less than 0.1%, making the content of effectively detected circRNA very low. Li et al. [47] revealed that circRNA microarray assay brings higher efficiency and sensitivity than RNA-seq for circRNA profiling. Unfortunately, microarray assays can only detect known circRNAs.

Through those two high-throughput technologies, accumulating evidence has disclosed that the dysregulated expression of circRNA is widespread in ESCC tissue and plasma. Utilizing circRNA microarray analysis, Song et al. identified 2046 differentially expressed circRNAs between 6 pairs of ESCC tissues and para-carcinoma tissues, of which 1148 were significantly up-regulated and 898 were down-regulated in ESCC tissues [48]. In another study, Sun et al. revealed 1055 differently expressed circRNAs with fold change ≥ 2, among which 418 of these circRNAs were up-regulated, and 637 were downregulated in ESCC tissues [49]. Jiang et al. [50] used RNA sequencing technologies to detect the differential expression of 3 pairs of ESCC tissue and resection margins. There is a total of 3288 circRNAs differentially expressed, including 2139 up-regulated, and 1149 down-regulated. Besides, in virtue of bioinformatics tools, the ceRNA network was constructed, and it revealed the possible mechanism and function of circRNAs in ESCC progression that could be further explored.

### CircRNAs mediate cancer cell proliferation

CircOGDH expression is higher in ESCC cells than in normal esophageal epithelial cells. Functional analysis indicated that circOGDH inhibits the negative effect of miR-615-5p on PDX1 expression, which leads to cell cycle inhibition [51]. Cell division cycle 6 (CDC6) is a critical DNA replication licensing factor, playing a pivotal role in the activation and maintenance of the cell cycle. CircNELL2, as an oncogenic factor, could activate the translation of CDC6 and promote cell proliferation through sponging miR-127-5p [52]. CircRIMS promotes miR-613 methylation to downregulate the expression of miR-613, thus resulting in cell proliferation promotion [53].

Besides, some circRNAs also act as tumor suppressors in ESCC progression. ITCH can negatively regulate the canonical Wnt pathway through degradation phosphorylated Dvl2 and arrest the oncogene c-Myc expression. Cir-ITCH, generated from ITCH, could enhance the expression of ITCH via sponging miRNAs such as miR-7, miR-17 and miR-214, thereby increasing c-Myc expression to induce cell proliferation and tumor growth [54]. Tumor suppressor gene Phosphatase and tension homolog (PTEN) is a superstar in multiple cancers development and progression, including ESCC. Activation of PETN could repress the PI3K/AKT signaling pathway to regulate many cellular actions, such as proliferation, cellular metabolism, differentiation, and apoptosis. Some circRNAs are found to regulate the PTEN expression in ESCC. For example, circ-Foxo3 has been observed to act as miR23-a sponge to elevate the expression of PTEN [55]. And circLAPR4 negatively modulated miR-1323 expression, thereby silencing PTEN/PI3K/AKT signaling pathway activity to hamper ESCC progression [56]. A novel circRNA, cCNTNAP3 exhibited a lower expression in ESCC and inhibited proliferation and increased apoptosis through miR-513a-5p/p53 axis. Notably, p53 also participated in the formation of cCNTNAP3, which formed a positive feedback loop to suppress ESCC development [57].

### CircRNAs involve in invasion and metastasis

EMT is a critical biological process that influences malignant tumor cells migration and invasion, featured by dysregulation of E-cadherin, N-cadherin, cytokeratin and Vimentin. Some signaling pathways related to circRNAs have been verified to participate in the EMT process, such as TGF-β and PI3K/Akt signaling axis. For instance, circLPAR3 was increased in ESCC and served as ceRNA to rescue c-MET suppression from miR-198. Increased expression of c-MET activated the phosphorylation of Akt and MAPK, thereby infecting the RAS/MAPK and PI3K/Akt pathways to stimulate cell invasion and migration [58]. He et al. verified that circVRK1 expression was decreased in ESCC, sponging for miR-624-3p, upregulating PTEN to restrain PI3K/AKT signaling pathway activity, and consequently boosting EMT [59]. Circ-NRIP1 is another circRNA that has been involved in PI3K/Akt pathway. This circRNA inhibits the negative effect of miR-595 on SEMA4D expression, which could reduce the restraint on PI3K/AKT signal [60]. Besides, circ-PRKCI could directly modulate Akt expression by acting as miR-3680-3p sponge [61]. In terms of TGF-β signaling pathway, circ-DOCK5 was identified to serve as a “reservoir” to stabilize the miR-627-3p expression. The amplified miR-627-3p arrests TGFB2, which can encode a ligand of TGF-β to interact with TGF-β receptors, thus involving TGF-β/SMAD/ZEB1 axis and suppressing the EMT process [62]. Altogether, circRNAs play a critical role in the EMT process through joining in regulating EMT-related pathways.

In addition, circRNAs also regulate cell migration and invasion via other pathways. CircNTRK2 was increased in ESCC tissue and involved in cell metastasis. This effect of circNTRK2 may be related to its interaction with miR-140-3p to weaken its suppression on E2F3 [63]. Another report revealed that circNTRK2 also sponge miR-140-3p to regulate the expression of NRIP1, bringing about the malignant cell behaviors of ESCC, including cell migration and invasion [64]. Liu et al. noticed that the expression level of serum exosomal hsa-circ0026611 was highly correlated with lymph node metastasis in ESCC, suggesting that circ0026611 may become a predictor of ESCC metastasis [65]. Knockdown of circRNA-100876 could significantly boost the expression of epithelial markers E-cadherin and attenuate the expression of mesenchymal markers N-cadherin and Vimentin [66]. The aboving results consistently validated the imperative role of circRNAs in tumor invasion and metastasis.

### CircRNAs regulate cell death

Abnormal regulation of programmed cell death is one of the hallmarks leading to carcinogenesis. A growing body of work showed that circRNAs performed an essential role in mediating cell apoptosis. Circ0120816 functions as a ceRNA of miR-1305 to reduce its inhibition of TXNRD1, which can attenuate the pro-apoptotic proteins including Cleaved PARP, Bax and Cleaved Caspase-3 to dampen ESCC cell apoptosis [67]. In addition, a study verified the close connection between circRNA and cell autophagy in ESCC. CiRS-7, interacting with miR-1299, strengthens EGFR level and inhibits starvation or rapamycin-induced autophagy of ESCC cells [68]. Pyroptosis, necroptosis and ferroptosis are novel discovered non-apoptotic programmed cell death mechanism, which can participate in tumor immune escape in tumor microenvironment and function in tumor progression as well as drug therapy [69]. CircPVT1 can obviously affect the expression of ferroptosis-related factors (GPX4 and SLC7A11) to enhanced chemoresistance in ESCC [70]. CircPUM1 is highly expressed in ESCC and can work to dampen pyroptosis progression to accelerate tumor growth [71]. However, at present the detail mechanism of this forms of cell death in ESCC remains open (Table 2).

**Table 2:** Function of circRNAs in ESCC

Name	Expression	Function	Sponge miRNAs	Target genes		Refs
circ-ITCH	↓	Inhibited proliferation	miR-7, miR-17, miR-214		ITCH	[54]
circCNTNAP3	↓	Inhibited proliferation and increased apoptosis	miR-513a-5p		TP53	[57]
circ- Foxo3	↓	Inhibited proliferation and accelerated cell apoptosis	miR-23a		PTEN	[55]
circLARP4	↓	Inhibited proliferation and accelerated cell apoptosis	miR-1323		PTEN	[56]
circVRK1	↓	Inhibited proliferation, migration, enhanced the sensitivity of ESCC cells to radiotherapy	miR-624-3p		PTEN	[59]
circ FAT1	↓	Inhibited migration and invasion	/		/	[72]
circ0071662	↓	/	/		/	[73]
circ-SMAD7	↓	Inhibited proliferation and migration	/		/	[74]
circ-DOCK5	↓	Inhibited migration and invasion	miR-627-3p		TGFB2	[62]
circPSMC3	↓	Increased the sensitivity of ESCC cells to n	miR-10a-5p		PTEN	[75]
cDOPEY2	↓	Inhibited cisplatin resistance	/		CPEB4/TRIM25	[76]
circIMMP2L	↑	Promoted proliferation, migration and invasion	/		CtBP1	[77]
circ_0014879	↑	Promoted proliferation, migration, invasion and radiosensitivity	miR-519-3p		CDC25A	[78]
circAGFG1	↑	Promoted proliferation, migration and invasion	miR-4306		MAPRE2	[79]
circ_0006168	↑	Promoted proliferation, migration, invasion and Taxol resistance	miR-194-5p1		JMJD1C	[80]
	↑	Promoted proliferation, migration and invasion	miR-516b-5p		XBP1	[81]
	↑	Promoted proliferation, migration and invasion	miR-384		RBBP7	[82]
	↑	Promoted proliferation, migration and invasion	miR-100		mTOR	[83]
	↑	Promoted proliferation and inhibited apoptosis	miR-384		FN1	[84]
circ_0072088	↑	Promoted proliferation, migration and invasion	miR-377		VEGF	[85]
circ_0004771	↑	Promoted proliferation, migration and invasion	miR-339-5p		CDC25A	[86]
	↑	Promoted proliferation, migration and invasion	miR-595		SEMA4D	[87]
circ0120816	↑	Promoted proliferation and inhibited apoptosis	miR-1305		TXNRD1	[67]
circ-ABCB10	↑	Promoted proliferation and invasion	miR-670-3p		/	[88]
circ-DLG1	↑	Promoted proliferation	/		/	[89]
circGSK3β	↑	Promoted migration	/		GSK3β	[90]
circRIMS	↑	Promoted proliferation	/		miR-613	[53]
circRNA_2646	↑	Promoted proliferation and migration	miR-124		PLP2	[91]
circRNA_141539	↑	Promoted proliferation and invasion	miR-4469		CDK3	[92]
circ- TTC17	↑	Promoted proliferation and migration	/		/	[93]
circ_0014715	↑	Promoted proliferation and migration	/		/	[94]
circLPAR3	↑	Promoted proliferation, migration and invasion	miR-375/miR-433		HMGB1	[95]
	↑	Promoted migration	miR-198		MET	[58]
circ-LRP6	↑	Promoted proliferation	miR-182		Myc	[96]
circNELL2	↑	Promoted proliferation	miR-127-5p		CDC6	[52]
circ_0087378	↑	Promoted proliferation, migration and invasion	miR-140-3p		NRIP1	[64]
	↑	Promoted proliferation, migration and invasion	miR-140-3p		E2F3	[63]
circ-PRKCI	↑	Promoted proliferation and migration	miR-3680-3p		AKT3	[61]
ciRS-7	↑	Promoted proliferation, migration and invasion	miR-876-5p		MAGE-A	[97]
	↑	Inhibited autophagy	miR‐1299		EGFR	[98]
	↑	Promoted migration and invasion	miR-7		KLF4	[99]
	↑	Promoted proliferation and migration	miR-7		HOXB13	[100]
circPDE3B	↑	Promoted proliferation, migration and invasion	miR-4766-5p		LAMA1	[101]
circ_0000654	↑	Promoted proliferation and migration	miR-149-5p		IL-6	[102]
circ_0000700	↑	Promoted proliferation and migration	miR-1229		/	[103]
circ-ZDHHC5	↑	Promoted proliferation, migration and invasion	miR-217		ZEB1	[104]
circRNA_100876	↑	Promoted proliferation, migration, invasion and inhibited apoptosis	/		/	[66]
circ_0012563	↑	Promoted migration and invasion	/		XRCC1	[105]
circR_100873	↑	Increased lymphatic metastases	/		/	[106]
circRNA6448-14	↑	Promoted proliferation, migration, invasion, and inhibited apoptosis	miR-455-3p		/	[107]
circ-0048117	↑	Promoted migration and invasion	miR-140		TLR4	[108]
circ_0003340	↑	Promoted proliferation, migration and invasion	miR-615-5p		PDX1	[51]
	↑	Promoted proliferation and inhibited cell apoptosis	miR-564		TPX2	[109]
circUBAP2	↑	Promoted proliferation, migration and invasion	miR-422a		Rab10	[110]
circ_0000337	↑	Promoted proliferation, migration and invasion	miR-670-5p		/	[111]
	↑	Promoted CDDP resistance	miR-377-3p		JAK2	[112]
circGOT1	↑	Promoted proliferation, migration, aerobic glycolysis, and cisplatin resistance	miR-606		GOT1	[113]
circ_0006948	↑	Promoted proliferation, migration and invasion	miR-3612		LASP1	[114]
	↑	Promoted proliferation, migration and invasion	miR-490-3p		HMGA2	[115]
circ_0023984	↑	Promoted proliferation, migration and invasion	miR-433-3p		REV3L	[116]
circDUSP16	↑	Contributed to hypoxia-stimulated ESCC cell progression	miR-497-5p		TKTL1	[117]
circRNA_100367	↑	Promoted radioresistant	miR-217		Wnt3	[118]
circ_0001093	↑	Promoted glutamine metabolism	miR-579-3p		glutaminase (GLS)	[119]
circCPSF6	↑	Promoted proliferation and inhibited cell apoptosis	/		CPSF6	[120]
circ_0007142	↑	Enhanced DDP-resistant	miR-494-3p		LASP1	[121]
circ_0003340	↑	Enhanced ESCC progression	miR-940		PRKAA1	[122]
circ-ATIC	↑	Enhanced ESCC progression	miR-326		ID1	[123]
circ_0000277	↑	Enhanced DDP-resistant	miR-873-5p		SOX4	[124]
circPUM1	↑	Regulated the mitochondrial oxidative phosphorylation	/		UQCRC2	[71]
circPVT1	↑	Inhibited 5-Fu sensitivity	miR-30a-5p		FZD3	[70]

### CircRNAs intervene in radioresistance and drug resistance

Chemotherapy and radiotherapy remain reliable strategies for ESCC patients to gain long-term survival. Prevailing radioresistance and drug resistance is one of the major obstacles resulting in poor prognosis of ESCC, but the mechanism of acquiring resistance is still unclear. Recently, many reports have confirmed that circRNAs regulated drug resistance by multiple processes. For instance, circVRK1 overexpression led to a significant activation of ESCC cells radiotherapy sensitivity [59]. Circ100367, an oncogenic circRNA, was extraordinarily responsive to the radioresistance of ESCC. The effect of circ100367-associated radioresistance depended on its significant activation of Wnt3 signaling pathway [118]. CircPSMC3 can bind to miR-10a-5p and prevent its inhibition of PTEN. Then, upregulation of circPSMC3 represses the sensitivity of ESCC cells to gefitinib [75]. Similarly, circ_0006168 was revealed to have roles in Taxol resistance in ESCC cells by regulating Jumonji domain containing 1C (JMJD1C) by sponging miR-194-5p [80]. Circ_0023984 was elevated in ESCC tissues and could associate with miR-433-3p and promoted REV3L expression [116]. Functionally, REV3L was confirmed to exert a negative effect in the regulation of sensitivity of ESCC cells to 5-fluorouracil [125], suggesting that circ_0023984 may influence the sensitivity to chemotherapy drugs. Further experimental investigations are needed to estimate the correlation between circRNAs and other chemotherapeutic reagents in ESCC.

### CircRNAs as diagnostic and prognostic biomarkers in ESCC

Early detection is the key to harbor successful therapies for tumor patients. It is now well established from various studies that many circRNAs may have the potential to become effective tumor biomarkers. As we mentioned above, circRNAs are associated with diverse pathological processes of ESCC. Besides, the characteristics of circRNAs endow them with evident advantages to be novel biomarkers in tumor diagnosis and prognosis. Firstly, circRNAs present high abundance, evolutionary conservation and own a longer half-life existing in exosomes and blood plasma. Meanwhile, they are expressed in a tissue-specific and developmental-stage-specific manner. With the development of RNA sequencing technology and bioinformatics analysis, circRNAs were easily detected and their roles in ESCC were extensively identified. Area under the curve (AUC) in the receiver operating curve (ROC) analysis is an evaluative criterion commonly used in circRNAs prediction performance. CircRNA-141539 and circRNA-6448–14 perform great diagnostic ability as their AUC in ESCC tissues is up to 0.81 and 0.91 [92, 107]. However, the stable existence and specific expression of circRNAs in the peripheral blood and exosomes have more value in diagnosis than tumor tissues. The plasma levels of circ-SLC7A5 and circ0004771 were significantly increased in ESCC patients with AUC at 0.77 and 0.82, showing their valuable diagnostic potential [86, 126]. Conversely, the expression of circ-SMAD7 is significantly decreased in ESCC tissue and plasma, and its AUC is 0.86, which demonstrates its diagnostic value [74]. Furthermore, combining different circRNAs or one circRNA with classical diagnostic biomarkers provides a novel idea to establish a more effective diagnostic system. The AUCs of has_circ_0001946 and hsa_circ_0062459 is 0.894 and 0.836, respectively. Fan et al. established a new formula to combine the expression level of hsa_circ_0001946 and hsa_circ_0062459 in plasma. And further ROC curve analysis showed that its AUC reaches 0.928, with 84% sensitivity and 98% specificity, giving a better diagnostic efficiency for ESCC [127]. CircGSK3β has been reported to be elevated in plasma and performed great value in the prognosis of ESCC as its AUC is 0.78. Combining with traditional biomarker CEA, the AUC reaches 0.80 [90].

Ample evidence indicates that circRNAs were closely related to the clinicopathological characteristics or prognosis of ESCC, such as TNM stage, lymph node metastasis, and overall survival (OS), which may play a key role in monitoring the prognosis of ESCC. Recent research showed that serum exosomal hsa_circ_0026611 expression was correlated with T stage, N stage, and postoperative radiotherapy and chemotherapy. And hsa_circ_0026611 can predict lymph node metastasis as a potential prognostic biomarker [65]. Similarly, the level of hsa_circ_0006948 in tissue was closely related to OS and lymphatic metastasis. The ROC curve analysis demonstrated its important value for predicting lymphatic metastasis [115]. These studies indicated that circRNAs had performed huge potential for ESCC diagnosis and prognosis. However, a larger cohort of clinical samples should be tested to verify the diagnostic and prognostic accuracy of circRNAs before applying to clinical practice.

### CircRNAs as therapeutic tools in ESCC

**Fig. 4:**
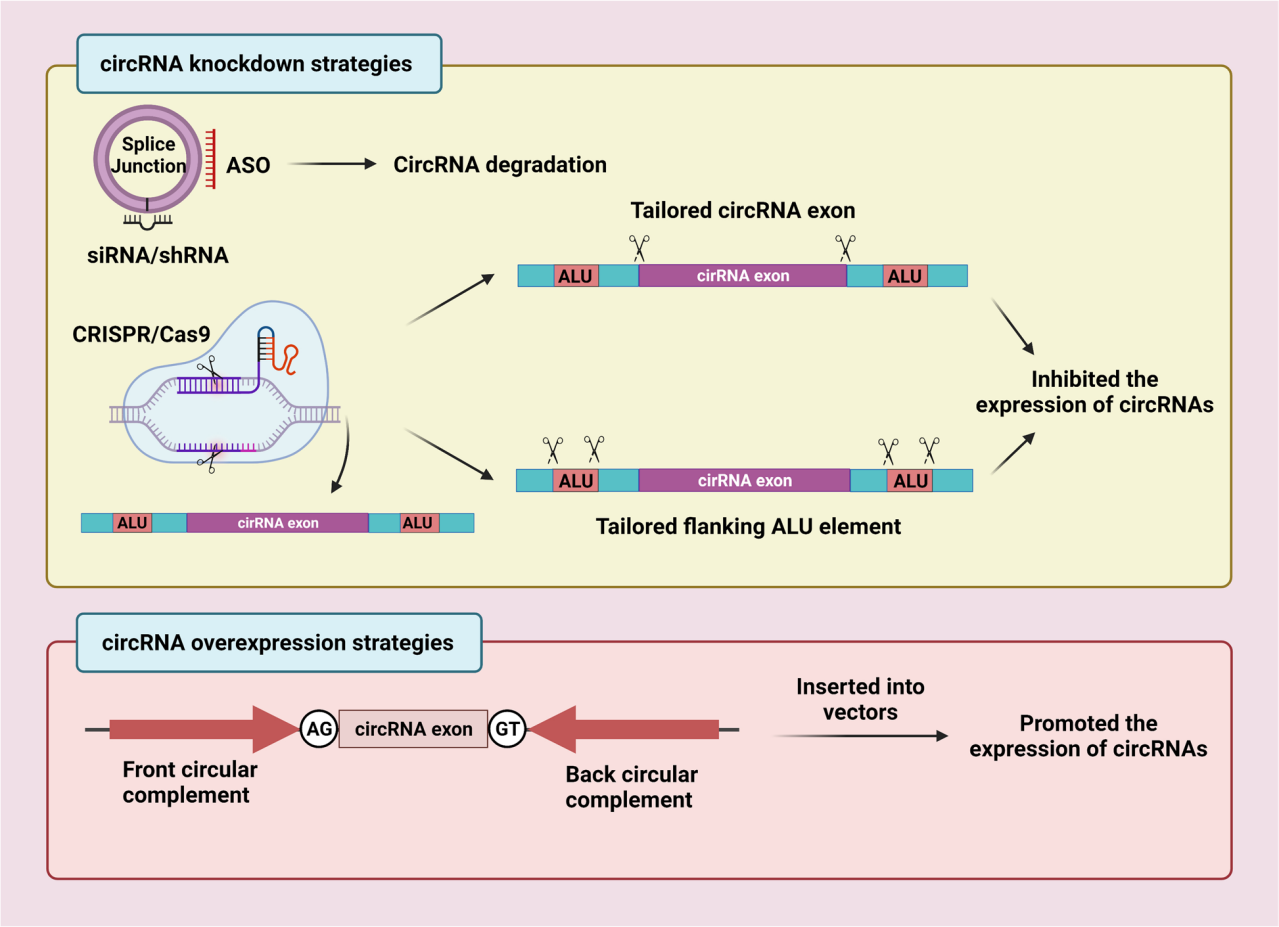
Summary of knockdown and overexpression strategies for circRNAs. Several therapeutic strategies based on the manipulation of circRNAs expression is expected to provide a brighter prospect for cancer therapy

The molecular pathogenesis of circRNAs involved in ESCC has been extensively revealed, circRNAs are proved to own the potential to be developed into promising and useful therapeutic targets. Knockdown of certain tumor-promoting circular RNAs or overexpression of some tumor suppressor circular RNAs can effectively reduce tumor volume, weight and block tumor metastasis in vivo. For instance, Zhou et al. identified that circPDE3B exhibits a tumor-promoting role in ESCC. CircPDE3B knockdown reduces tumor growth and the number of lung micrometastatic nodules [101]. As a tumor suppressor, the cCNTNAP3 expression level is downregulated in ESCC cell lines, while overexpression of cCNTNAP3 efficiency attenuated tumor growth [57]. Several manipulation methods of circRNAs expression have been applied (Fig. 4). RNA interference (RNAi) is commonly used in circRNAs knockdown, including antisense oligonucleotides (ASO) and siRNA or shRNA technology [128]. They can specifically combine with backspliced junction to induce gene silencing. In addition, CRISPR/Cas13 system can acquire complete removal of circRNAs through two different approaches: the deletion of the circRNA coding genome locus [129] or erasing of flanking ALU elements [11]. Compared with RNAi, CRISPR/Cas13 system was characterized by higher knockdown efficiency and specificity, becoming a promising method in RNA silencing research. However, owing to the unknown side-effects delivered by the exogenous Cas13 protein and guide RNA, whether CRISPR/Cas13 is adaptable to clinical needs still needs to be further studied [130]. Intron-pairing-driven circularization is a classical strategy for the construction of circRNAs overexpression vectors. The circRNA sequence was inserted into vectors (plasmid, lentivirus, AAV vector, etc.) containing reverse complement sequence for hybridization. Also, as an essential function of circRNAs, miRNA sponge can lead to miRNA acting as oncogenes loss-of-function, performing colossal potential to become a powerful molecular therapeutic strategy. Wang et al. constructed a circRNA that could repress endogenous miR-21 and miR-93. This synthetic miRNA sponge performed tumor-suppressive effects in vitro and in vivo, restraining ESCC cell proliferation, migration, and tumor growth [131]. Compared to linear RNAs, high stability and reduced immunogenicity enabling exogenous circRNAs more likely to be delivered safely in vivo. However, research on circRNAs as treatment strategies is still at an early stage, and there exist many unresolved problems. Evaluating safety is the foremost concern before clinical practice. Additionally, whether circRNAs can successfully apply to clinical practice mainly depend on the precise delivery of synthetic circRNAs. How to deliver circRNAs safely into the proximity of tumor lesions to retard cancer progression demand prompt solution. Taken together, the characteristics and functions of circRNAs confer great potential for future molecular targeting treatment and the application of circRNA-based therapeutic strategies.

## Conclusions and perspectives

As the newly emerging endogenous ncRNAs, circRNAs are being given more attention and have become a hot spot in cancer research. CircRNAs generated from pre-mRNAs back-splicing, they are conserved, abundant, stable, and considered as important regulators of multiple physiological and pathological processes. The rapid advances of high-throughput detection technology have allowed a large number of differentially expressed circRNAs to be detected in ESCC tissues. As described above, many studies on circRNAs related to ESCC have provided convincing evidence that circRNAs participate in ESCC cell proliferation, migration, invasion, apoptosis, and therapy resistance. CircRNAs perform highly specifically roles in tissues and cells, making them have the opportunity to become valuable prognostic and diagnostic biomarkers for ESCC. Furthermore, the unique properties of circRNAs on conformation, stability and immunogenicity will be of interest to explore RNA circle-based technologies, which include regulating innate immune responses, serving as sponges of cellular miRNAs and as aptamers to interfere with intracellular processes [132]. The research on those innovative technologies may open up a novel field in diagnosis and treatment of many diseases.

A growing body of reports underlines the importance of circRNAs in ESCC and its strong correlation with ESCC pathophysiological feature. And a sea of innovative research on the application of circRNAs is already in full swing. However, not a single circRNA-based medical application has been approved so far. Compared with miRNA and lncRNA research, circRNAs research is still at its sunrise and has many challenges and problems need to be solved. For instance, the mechanism of circRNAs biogenesis, translocation and degradation is still largely unknown. More comprehensive understanding of this mechanism may help the development of novel technologies. Additionally, reports on the functions of circRNAs in ESCC mainly focused on miRNA sponges, but the function of binding protein and coding potential of circRNAs still lags behind. In fact, only a small portion of circRNAs potential capability have been identified, so it is imperative to further elucidate whether there are other unknown effects. How to facilitate the translation of circRNAs research to the clinical setting is the ultimate goal at present. Firstly, novel technologies and detection methods are required to settle the problem of circRNAs low abundance in biological samples and further improve the quality and precision of detection. Besides, their application as noninvasive biomarkers will need a large pool of tumor samples for verification. Studies can focus on building combined detection methods to gain better diagnostic value. At last, more clinical statistics are needed to build the appropriate collection methods and cutoff values of specific circRNAs in tumor diagnosis. Looking forward, despite the limitations mentioned above, with the explosion in RNA circle-based technologies and in-depth understanding of the features and functions of circRNAs, this type of non-coding RNAs will unveil its translational relevance in ESCC prevention, diagnosis and treatment.

## Data Availability

Not applicable.

## Abbreviations

ESCC: Esophageal Squamous Cell Carcinoma
ECA: Esophageal Adenocarcinoma
circRNAs: Circular RNAs
ncRNAs: Non-coding RNAs
RNA-seq: RNA sequencing
pre-mRNAs: Precursor mRNAs
EcRNAs: Exonic circRNAs
EIciRNAs: Exon–intron circRNAs
ciRNAs: Circular intronic RNAs
RBPs: RNA binding proteins
pre-tRNAs: Precursor tRNAs
tricRNAs: Circular RNAs
TSEN: TRNA splicing endonuclease
BHB: Bulge-helix-bulge
QKI: Quaking
miRNAs: MicroRNAs
MREs: MiRNA response elements
ceRNA: Competitive endogenous RNA
Sry: Sex-determining region Y
IRES: Internal ribosome entry site
m6A: N6-methyladenosine
FUBP1: Far upstream element binding protein 1
CDKN1: Cyclin-Dependent Kinase Inhibitor 1
CDC6: Cell Division Cycle 6
PTEN: Phosphatase and Tension Homolog
EMT: Epithelial-to-Mesenchymal Transition
JMJD1C: Jumonji Domain Containing 1C
AUC: Area Under the Curve
ROC: Receiver Operating Curve
OS: Overall Survival
RNAi: RNA interference
ASO: Antisense Oligonucleotides
